# Neuronal ceroid lipofuscinosis type 5 in Russia: first case report and literature review

**DOI:** 10.3389/fmed.2025.1581597

**Published:** 2025-08-25

**Authors:** Olga P. Parshina, Anastasiia A. Buianova, Svetlana V. Mikhaylova, Sergey V. Piliya, Alikhan A. Alikhanov, Elena K. Donyush, Zinaida A. Kondrashova, Nadezhda V. Liakhova, Oleg N. Suchalko, Alina F. Samitova, Anna O. Shmitko, Mayya V. Zazhivikhina, Natalya A. Votyakova, Dmitriy O. Korostin

**Affiliations:** ^1^Genomics Laboratory, Institute of Translational Medicine Pirogov Russian National Research Medical University, Moscow, Russia; ^2^Russian Children’s Clinical Hospital, Moscow, Russia

**Keywords:** neuronal ceroid lipofuscinosis, epilepsy, CLN5, neurodegenerative disease, next-generation sequencing

## Abstract

Neuronal ceroid lipofuscinosis (NCL) is one of the most common causes of childhood dementia. NCL type 5 is characterized by epileptic seizures, cognitive decline, and progressive vision loss. Whole exome sequencing was performed, and the identified variant was confirmed by Sanger sequencing. Despite various therapeutic regimens, including novel approaches, seizure control could not be achieved. The disease was caused by a previously undescribed pathogenic variant *CLN5*(NM_006493.4):c.368del (p.Arg123LysfsTer4). This is the first known case of NCL type 5 in Russia. Unusually, the patient also had a cervical lymphangioma requiring separate medical and surgical intervention. This case report contributes to our understanding of the natural history of CLN5-associated NCL and may support the development of gene therapy approaches for its treatment.

## 1 Introduction

Neuronal ceroid lipofuscinosis (NCL) is a group of neurodegenerative disorders primarily affecting children. A hallmark of NCL is the accumulation of autofluorescent lipofuscin-like storage material in body tissues (1). The global incidence is estimated at 1–8 per 100,000 individuals (2, 3), making NCL the leading cause of childhood dementia (2). There are no current epidemiological data on NCL type 5 in Russia. Preliminary analyses indicate that pathogenic, likely pathogenic, and variants of uncertain significance (VUS) in the *CLN5* gene are exceptionally rare, with only six VUS found among over 6,300 individuals in a Russian control cohort (3).

Biallelic variants in at least 13 genes have been implicated in autosomal recessive forms of NCL. Clinical features include progressive vision loss, epilepsy, and cognitive and motor decline. However, initial manifestations vary: some NCL subtypes present first with visual impairment, while others begin with seizures (4). Notably, all NCL forms are progressive and currently lack disease-modifying treatments (5), except CLN2 disease, where cerliponase alfa – a recombinant form of tripeptidyl peptidase 1 (TPP1) that replaces the deficient enzyme – has demonstrated significant efficacy in slowing disease progression (6). Here, we describe a patient with a novel pathogenic variant in the *CLN5* gene who presented without visual impairment, thus broadening the phenotypic spectrum and contributing to the natural history of CLN5 disease. We also provide a literature review to contextualize this case within the current understanding of neuronal ceroid lipofuscinosis type 5.

## 2 Materials and methods

### 2.1 Clinical methods and DNA diagnostics

The patient was under continuous medical supervision at the Department of Hematology and Chemotherapy, Russian Children’s Clinical Hospital (RCCH), starting at 5 months of age. The patient also received follow-up care from the Otorhinolaryngology Department. Beginning at age 7.5 years, the patient was monitored by the Department of Psychoneurology, and from age 8, by the Department of Medical Genetics. The patient underwent regular multidisciplinary evaluations at 6-month intervals throughout the disease course. Magnetic resonance imaging (MRI) was performed using a GE Discovery 750w scanner in three orthogonal planes under general anesthesia. The imaging protocol included an axial T2-weighted (TR 6198 ms, TE 108.864 ms), an axial FLAIR (TR 10,500 ms, TE 140 ms), and sagittal T1-weighted (TR 923 ms, TE 12.392 ms) sequences. Electroencephalographic (EEG) recordings were acquired using the Encephalan video-EEG monitoring system (Medicom MTD, Russia) with electrodes positioned according to the international 10–20 system.

Whole exome sequencing (WES) of genomic DNA extracted from the patient’s peripheral blood sample was carried out using the Agilent SureSelect Human All Exon V8 baits (Agilent Technologies, Santa Clara, CA, USA) at the Genomics laboratory, Institute of Translational Medicine, Pirogov Russian National Research Medical University, following the RSMU_exome protocol (7). DNA libraries were prepared using the MGIEasy Universal DNA Library Prep Set (MGI Tech, China), followed by target enrichment and paired-end sequencing (PE100) on the DNBSEQ-G400 platform, achieving 100 × mean coverage. Raw sequence data quality was evaluated using FastQC v0.11.9 (8). Sequencing quality reports identified imbalanced bases at read starts, which were trimmed using BBDuk (BBMap v38.96) (9). Processed reads were then aligned to the GRCh38.p14 reference genome assembly employing bwa-mem2 v2.2.1 (10), with subsequent file format conversion and coordinate sorting implemented through SAMtools v1.9 (11). Duplicates were marked using Picard v2.22.4 (12). Variant calling was executed through a dual approach incorporating bcftools mpileup v1.9 (13) and DeepVariant v1.5.0 (14), followed by variant decomposition into biallelic representations using vt decompose v0.5772 (15) and normalization via vt normalize v0.5772. Filtering criteria included coverage depth ≥3 and FILTER = PASS for DeepVariant. The resultant VCF files were merged using bcftools-1.9 and annotated via ANNOVAR (16). Clinical interpretation adhered to ACMG guidelines, incorporating minimum coverage thresholds (14×) and population frequency cutoffs (gnomAD v4.1.0 < 0.01), with additional consideration of Russian-specific allele frequencies derived from the RUSeq browser and Database of Population Frequencies (17–21). The following phenotype-based panels were used: ‘Abnormal bleeding’ HP:0001892, ‘Hyperlipidemia’ HP:0003077, ‘Lymphedema’ HP:0001004, ‘Seizure’ HP:0001250 (22). No pathogenic, likely pathogenic, or VUS variants were detected in the first three panels. The *CLN5* mutation was subsequently confirmed in all family members using Sanger sequencing. Primers were designed with Primer3; the forward primer: 5′-GGGTTGGGAGTGAG TGACTG-3′, and the reverse primer: 5′-CTTGCTGGTGTGACC CCTTA-3′.

### 2.2 Literature review

A systematic literature review was conducted in PubMed in October 2024 using the search terms: ‘neuronal ceroid lipofuscinosis’, ‘neuronal ceroid lipofuscinosis type ‘, ‘neuronal ceroid lipofuscinosis type 2′, and *‘CLN5’*. Inclusion criteria restricted selection to English-language publications with at least one search term appearing in the title. From the identified records, we selected 15 of the most relevant and recent publications providing comprehensive coverage of disease characteristics, including clinical manifestations, pathogenesis, diagnostic approaches, and treatment strategies. For clinical analysis, the inclusion criteria required precise HGVS variant nomenclature, along with either EEG data or detailed patient clinical descriptions. The characteristic disease phenotype was delineated through analysis of three studies (total n = 24 patients) combined with our case observations. To evaluate therapeutic approaches, we expanded the search strategy by adding the terms ‘treatment’, ‘seizures’, and ‘antiepileptic therapy’. Importantly, none of the identified treatment approaches showed significant efficacy.

## 3 Results

### 3.1 Case description

This case is notable for the coexistence of a giant cervical-facial lymphangioma and NCL, a rare combination. Initially, delays in motor and cognitive development were attributed to complications resulting from extensive lymphatic malformation treatment, including tracheostomy, multiple surgical interventions, cytostatic therapy, and prolonged hospitalization. However, the development of ataxia and the regression of previously acquired skills raised concerns. These findings, supported by MRI, prompted further diagnostic evaluation to investigate the underlying causes of epilepsy and neurodegeneration. WES revealed a previously undescribed homozygous pathogenic variant, *CLN5*(NM_006493.4):c.368del (p.Arg123LysfsTer4).

The proband is a 9-year-old male, born to non-consanguineous, clinically healthy parents. He has a 5-year-old sister who is also clinically healthy, and the family history is unremarkable. The patient was born at term via cesarean section following an uneventful pregnancy, with a birth weight of 4,200 g and a length of 52 cm. The neonate presented with an extensive combined lymphatic-venous malformation involving multiple anatomical regions: the floor of the mouth, submental region, cervical area, bilateral buccal regions, and lingual surface. Due to upper airway obstruction causing respiratory distress, he required neonatal intensive care unit admission and was discharged on the sixth day of life.

At 3 months of age, a tracheostomy was performed. For 5 months, he has been under regular observation at the RCCH. His treatment included intermittent administration of sirolimus (Rapamune®) and co-trimoxazole (Biseptol®) for Pneumocystis pneumonia prophylaxis. While clinical improvement was inconsistent, it was overall positive. He also developed laryngeal stenosis.

Despite a global neuropsychological developmental delay, gross motor milestones were initially within expected limits: head control was achieved by 2 months, independent sitting by 5–6 months, walking by 15 months, and syllable production by 18 months. By age 3, the child was using short phrases. However, at age 4, speech regression began, including dysarthria and marked vocabulary loss. Hamburg Scale (the Clinical Scoring System for Late Infantile Neuronal Ceroid Lipofuscinoses (LINCL)) was 11 at this stage (Table 1).

**Table 1:** Evaluation of the patient’s clinical status according to the Hamburg Scale for Late Infantile Neuronal Ceroid Lipofuscinoses (LINCL) (30).

Functional category	Performance and score	4 years	5 years	6 years	7 years 6 months	8 years 3 months	9 years
Motor function	Walks normally^a^ – 3 Frequent falls, clumsiness obvious – 2 No unaided walking or crawling only – 1 Immobile, mostly bedridden – 0	3	3	2	2	1	0
Language	Normal (individual maximum)^b^ – 3 Has become recognizably abnormal – 2 Hardly understandable – 1 Unintelligible or no language – 0	2	2	2	2	1	1
Visual function	Recognizes desirable object, grabs at it – 3 Grabbing for objects uncoordinated – 2 Reacts to light – 1 No reaction to visual stimuli – 0	3	3	3	3	3	3
Seizures (grand mal)	No seizure per 3-mo period – 3 1 to 2 seizures per 3-mo period – 2 1 seizure per mo – 1 >1 seizure per mo – 0	3	3	2	0	0	0
Score (0–12)		11	11	9	7	5	4

^a^ Some children never attained normal motor function.

^b^ Some patients never developed age-appropriate language skills.

**Figure 1:**
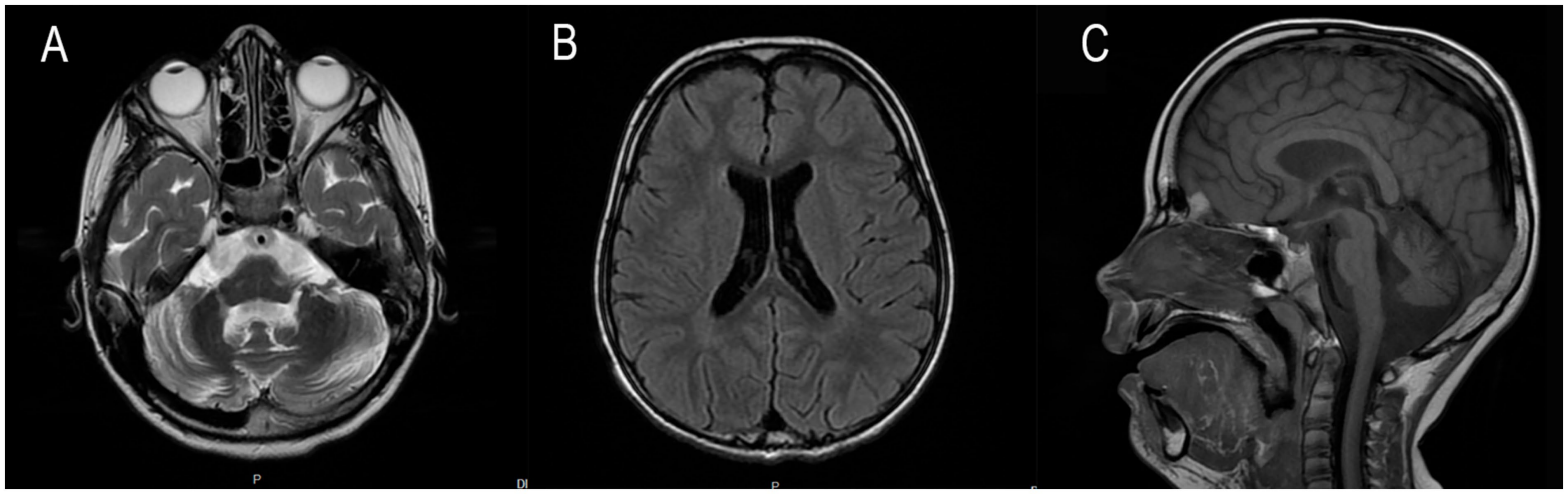
**(A)** Axial T2-weighted slice demonstrates excessive widening of cerebellar sulci and expansion of cisternal spaces in the posterior cranial fossa, reflecting moderate cerebellar atrophic volume loss. **(B)** Axial T2 FLAIR slice reveals mild periventricular leukoencephalopathy in the white matter of the frontal and parietal lobes. **(C)** Sagittal T1-weighted slice shows reduced cerebellar volume with an enlarged posterior cranial fossa, clearly illustrating cerebellar atrophy.

At the age of 5, the patient underwent a brain MRI to evaluate the feasibility and scope of potential surgical intervention. Imaging revealed cerebellar volume loss (Figure 1A) and periventricular leukoencephalopathy in the frontal and parietal white matter (Figure 1B). These findings indicated that the observed developmental regression and seizures were unlikely to be related to functional neurological disturbances or perinatal hypoxic–ischemic injury, but instead pointed toward a lysosomal storage disorder. Hamburg Scale for LINCL was 11 at that time. A neck mass was surgically excised, with histological confirmation of lymphatic malformation.

At the age of 6, the patient began experiencing epileptic seizures, manifesting as gaze fixation accompanied by tonic stiffening of the limbs, lasting several seconds. The seizures, combined with progressive loss of motor and speech skills and the development of ataxia, strongly suggested a hereditary form of epilepsy (Table 1). The Hamburg Scale for LINCL score subsequently declined to 9.

**Figure 2:**
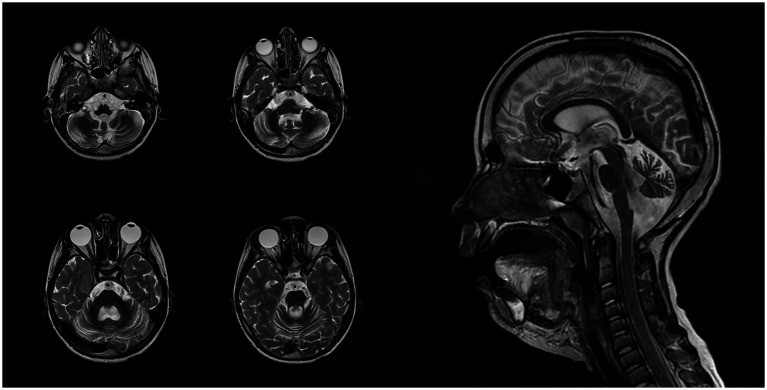
Magnetic resonance imaging of the patient’s brain performed in 7 years 6 months ago.

At 7 years and 6 months, a follow-up brain MRI with intravenous contrast revealed progression of cerebellar atrophy, cerebral subatrophy, and lateral ventricular dilatation measuring up to 14 mm (Figure 2). Mild leukoencephalopathy persisted in the periventricular parietal white matter. No displacement of midline structures was observed, and the subarachnoid spaces remained within normal limits. The hippocampal, parahippocampal, and hypothalamic–pituitary regions appeared structurally intact, and the craniovertebral junction was normally formed. No pathological contrast enhancement was observed. Overall, sequential imaging demonstrated stable disease without significant progression over time.

**Figure 3:**
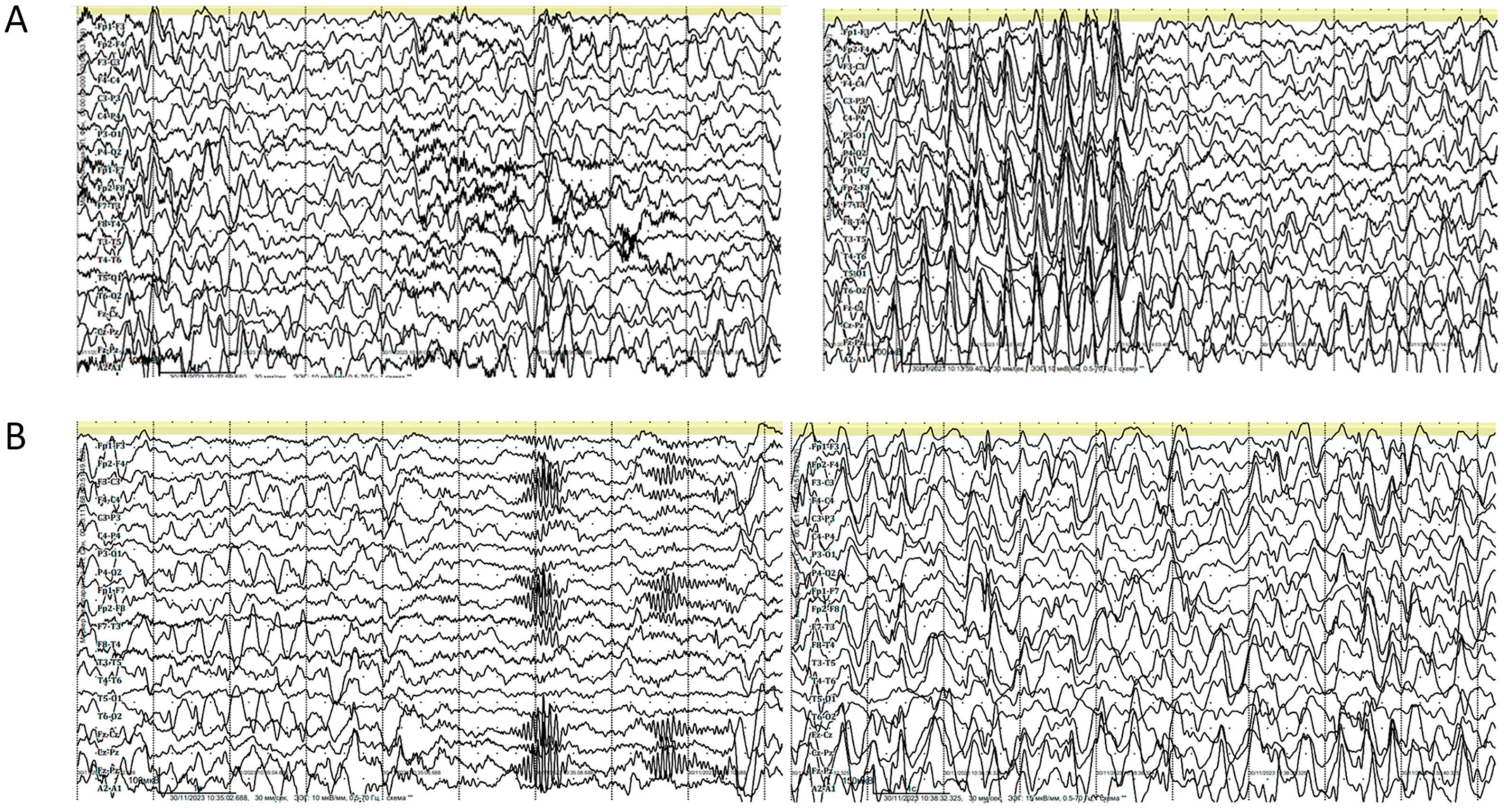
**(A)** Electroencephalography (EEG) during wakefulness (age 7 years and 6 months). Parameters: speed = 30 mm/s, sensitivity = 10 μV/mm, high-frequency filter = 0.5 Hz, low-frequency filter = 70 Hz, notch filter = 50 Hz, bipolar longitudinal montage. **(B)** EEG during daytime sleep (age 7 years and 6 months). Parameters: speed = 30 mm/s, sensitivity = 10 μV/mm, low-frequency filter (LFF) = 0.5 Hz, high-frequency filter (HFF) = 70 Hz, notch filter = 50 Hz, bipolar longitudinal montage.

EEG at this age revealed abnormalities during both wakefulness and sleep. The wakefulness EEG (Figure 3A) showed cortical rhythms that were markedly disorganized and inappropriate for the patient’s age, with diffuse epileptiform activity. Grouped spike–wave complexes were recorded with bioccipital and bifrontal distribution. Additionally, regional epileptiform activity was recorded independently in the right parieto-occipital and left parieto-central regions, frequently exhibiting diffuse spread. The epileptiform activity index fluctuated between low and high values, averaging above normal. No pathological changes were elicited by functional provocation. A generalized atonic seizure was captured during clinical observation, with a corresponding diffuse ictal EEG pattern.

EEG recordings during daytime sleep and wakefulness (Figure 3B) revealed cortical rhythms that were inconsistent with the patient’s age, exhibiting pronounced disorganization and epileptiform activity. Sleep stages were well differentiated, with physiologically distinct stages. Independent multiregional epileptiform discharges included: grouped spike–wave complexes with posterior projection; bifrontal grouped spike–wave activity; and regional discharges in the right parieto-occipital area, frequently extending to temporal leads (O2, P4 → T6, T4). The wakefulness index remained within the average range. During sleep onset, the epileptiform activity index initially decreased but subsequently intensified, showing variable levels from low to high, with an overall average range throughout the recording. No epileptic seizures or pathological EEG patterns were detected during this session, and no clinical seizures occurred during monitoring.

At age 8, an ultrasound examination of the soft tissues of the lower jaw revealed diffuse hyperechoic changes with a microcystic structure and poorly defined boundaries. The venous pattern in the lower lingual region was dilated, though without active blood flow. Anechoic inclusions up to 4 mm in size were identified in the right submandibular area. The patient continues to receive sirolimus (1.5 mg/day), a drug known to cause elevated serum lipoprotein levels, which were observed in this case (23).

**Figure 4:**
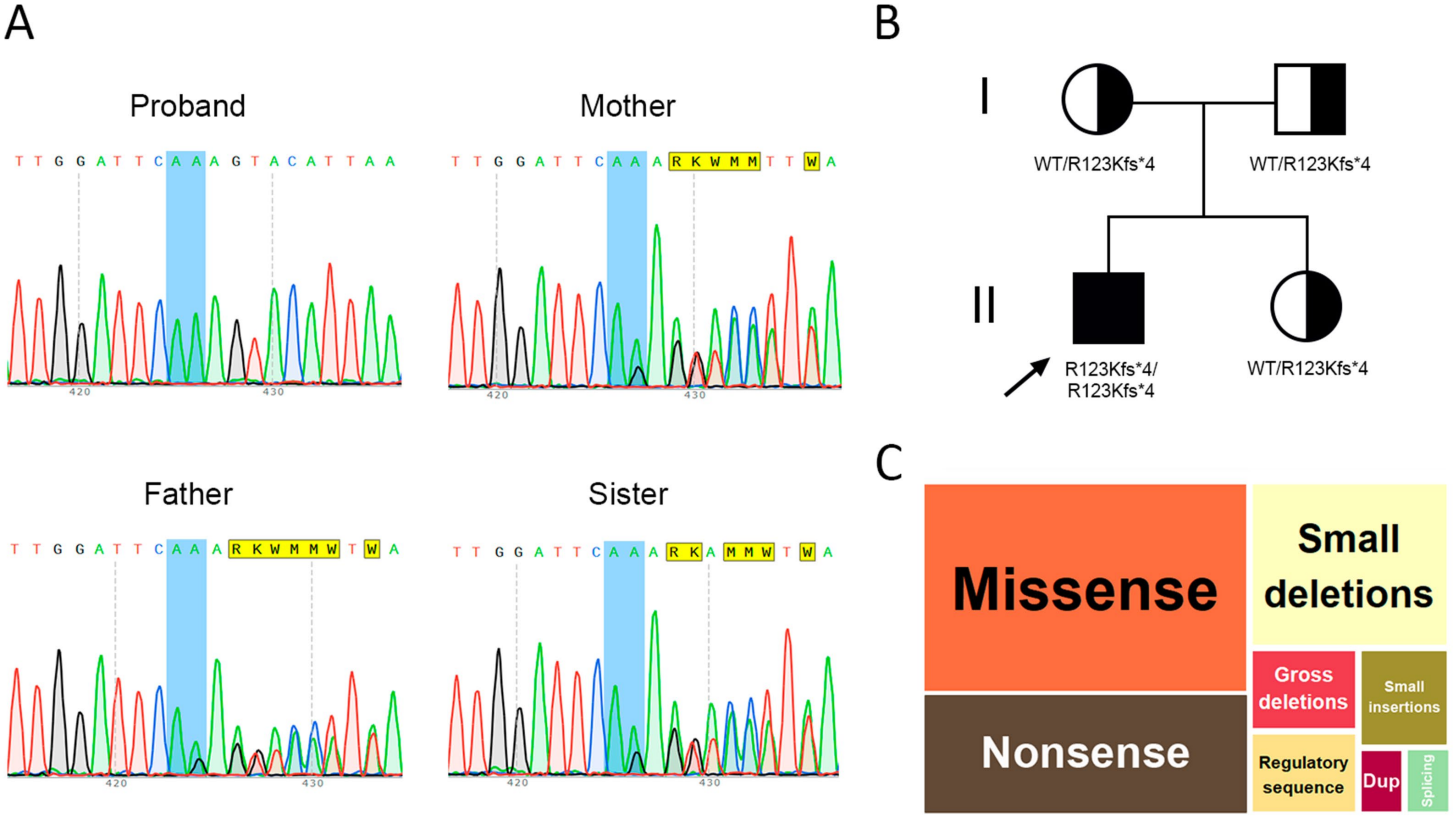
**(A)** Sanger sequencing results in the nuclear family, **(B)** Pedigree of the proband, **(C)** Distribution of variants in the *CLN5* gene by type, dup – duplication.

WES was recommended to clarify the etiology of the patient’s condition. Variant filtering using the ‘Seizure’ phenotype panel (HP:0001250) identified a homozygous deletion: chr13:76995929AG > A, corresponding to *CLN5*(NM_006493.4):c.368del (p.Arg123LysfsTer4). This variant is classified as pathogenic based on ACMG criteria (PVS1, PM2, PM3). Segregation analysis and variant confirmation were performed by Sanger sequencing in the proband, his parents, and his younger sister. The proband was found to be homozygous for the variant, while both parents and the sibling were heterozygous carriers (Figures 4A,B).

Over the past 6 months, the patient has experienced further neurological decline, including the complete loss of independent ambulation. Nevertheless, his hearing and vision remain preserved. At 8 years of age, the patient demonstrated comprehension of spoken language and retained a limited expressive vocabulary of two words. He was, however, unable to perform self-care. Epileptic seizures persisted, manifesting as episodes of upward gaze lasting approximately 10 s, with a frequency of 10–15 episodes per day. Longitudinal observation revealed a 1-point decrease on the Hamburg LINCL Scale. On the one hand, a positive trend was noted, with seizure frequency reduced to 4–5 episodes per day. On the other hand, the child lost the ability to walk independently. The most effective seizure control was achieved through a combination therapy of valproic acid, benzodiazepines, and levetiracetam – an approach supported by existing literature (24).

Following epilepsy onset at age 6, the patient was initially treated with age-appropriate doses of zonisamide and valproic acid (350 mg twice daily). At 7 years and 6 months, valproic acid was transitioned to an extended-release formulation at 450 mg twice daily, with zonisamide continued at 250 mg twice daily. At age 8, clonazepam was introduced but proved ineffective and was discontinued after 2 weeks. The current regimen consists of extended-release valproic acid granules (300 mg at 8:00 AM and 500 mg at 8:00 PM), levetiracetam (750 mg twice daily), and clobazam (2.5 mg twice daily). While a reduction in seizure frequency and duration was achieved, complete control of seizure activity was not attained.

The treatment history is summarized in Table 2, and serial Hamburg LINCL Scale scores are presented in Table 1.

**Table 2:** Treatment timeline of the patient with Neuronal Ceroid Lipofuscinosis (NCL) type 5.

Patient’s age	Intervention and outcome
The first day of life	A combined lymphatic-venous malformation involving the oral floor, chin, neck, cheeks, and tongue was diagnosed. Due to upper airway obstruction causing respiratory distress, the patient was transferred to the neonatal intensive care unit (NICU).
6 days of live	The patient was discharged from the NICU.
3 months – present	A tracheostomy was performed, and the patient is currently tracheostomy-dependent.
5 months - present	The patient has been receiving sirolimus (Rapamune®) and co-trimoxazole (Biseptol®). Although administered irregularly, this regimen has shown clinical benefit. The patient subsequently developed laryngeal stenosis.
18 months	Emergence of the first syllables.
3 years	Use of short phrases.
4 years	Onset of dysarthria and significant regression in expressive vocabulary. Hamburg LINCL scale score: 11
5 years	Surgical excision of a cervical mass was performed; histological analysis confirmed lymphatic malformation. Hamburg LINCL scale score: 11
6 years	The first epileptic seizures appeared, characterized by gaze fixation and tonic limb stiffening lasting several seconds. The patient was treated with zonisamide at age-appropriate doses and valproic acid at 350 mg twice daily. This regimen proved to be effective. Hamburg LINCL scale score: 9
7 years and 6 months	Whole exome sequencing was performed for the patient, and Sanger sequencing was also conducted for his family. The patient received extended-release valproic acid (450 mg twice daily) and continued zonisamide (250 mg twice daily). Hamburg LINCL scale score: 7
8 years and 2 months	Progressive motor decline led to complete loss of ambulation. Although receptive language remained intact, expressive language was reduced to two words, and the patient became fully dependent on self-care. Seizures occurred 2–5 times daily. A trial of clonazepam for 2 weeks was ineffective.
8 years and 3 months	Treatment was modified to valproic acid in extended-release granules (300 mg at 8:00 AM and 500 mg at 8:00 PM), levetiracetam (750 mg twice daily), and clobazam (2.5 mg twice daily). This regimen resulted in a noticeable reduction in seizure frequency and duration. Hamburg LINCL scale score: 5
9 years	The patient remains on the established treatment regimen. There has been a partial positive response, with seizure frequency reduced from 10–15 episodes per day to 4–5 episodes per day. Hamburg LINCL scale score: 4

The first MRI was performed at the age of 5 years (Figure 1). An axial T2-weighted image demonstrated pronounced widening of the cerebellar sulci and enlargement of the cisternal spaces in the posterior cranial fossa, indicative of moderate cerebellar volume loss consistent with cerebellar atrophy (Figure 1A). An axial T2-FLAIR image revealed mild periventricular leukoencephalopathy involving the white matter of the frontal and parietal lobes (Figure 1B). A sagittal T1-weighted image showed reduced cerebellar volume and expansion of the posterior cranial fossa, clearly illustrating cerebellar atrophy (Figure 1C). A follow-up MRI was conducted at the age of 7 years and 6 months due to progression of neurological symptoms (Figure 2). From a dynamic perspective over the observed interval, no significant changes were noted compared to the previous imaging. An EEG performed at 7 years and 6 months during wakefulness (Figure 3A) revealed cortical rhythms inconsistent with the patient’s age, characterized by generalized disorganization and epileptiform discharges. Daytime EEG recordings during both wakefulness and sleep (Figure 3B) confirmed markedly disorganized cortical activity with prominent epileptiform features. WES identified a homozygous variant: chr13:76995929AG > A, *CLN5*(NM_006493.4):c.368del (p.Arg123LysfsTer4), which was classified as pathogenic according to ACMG criteria (PVS1, PM2, PM3). Sanger sequencing confirmed the variant and the patient’s homozygous status.

Regarding the literature review, we provided a detailed description of the typical pathogenesis and characteristic clinical features of the disease, which were then compared with the findings in our proband. A comparative table was compiled summarizing the clinical characteristics of patients reported in selected studies. In addition, we performed a population analysis of variant prevalence, including data from Russia, and assessed the representation of specific SNPs within the *CLN5* gene (Figure 4C).

### 3.2 Results of literature review

NCL type 5 is caused by biallelic pathogenic variants in the *CLN5* gene. This disorder is characterized by progressive neurodegeneration manifesting as ataxia, regression of motor and cognitive functions, epilepsy, and progressive vision loss, including macular degeneration. The typical age at death ranges from 13 to 30 years (5). Early clinical signs include speech and cognitive delays, followed by the onset of epileptic seizures 3 to 4 years later (25). In the present case, initial symptoms began at the age of four, with delays in speech and cognitive development, and epilepsy developed by age six. Seizures subsequently became pharmacoresistant.

Neuroimaging findings in NCL5 may include cerebellar atrophy, hypointensity of the thalamic nuclei, and hyperintensity in the periventricular white matter and internal capsule (5) – features all observed in this patient. EEG reveals occipital lobe epileptiform activity in response to photic stimulation at low frequencies (5). However, epilepsy in NCL type 5 usually begins later than in other NCL forms, between the ages of 7 and 11 years, most often presenting as myoclonic seizures. Additionally, NCL type 5 is associated with a gradual slowing of background EEG activity accompanied by epileptiform discharges, such as spikes, spike–wave complexes, and polyspikes. Retinal degeneration, detectable by electroretinography, typically manifests between the ages of 6 and 10 (2), was notably absent in this case, underscoring the diagnostic value of this clinical observation. Histopathological studies in NCL5 demonstrate lipofuscin accumulation with characteristic granular and ‘fingerprint’ profiles (5). Cortical atrophy is considered a key contributor to the disease’s pathogenesis (26).

Several functionally confirmed pathogenic variants have been reported. For example, a homozygous *CLN5*:c.434G > C (p.Arg96Pro) variant initially classified as a VUS was subsequently proven causative (27). Most known *CLN5* variants are loss-of-function, including p.Gly128Trpfs10, p.Arg150*, p.Trp175*, p.Trp330*, and p.Tyr343*. Pathogenic missense variants have also been described (e.g., p.Val214Glu, p.Tyr209Asp, p.Arg63His, p. Trp158Arg) (25, 28). According to the Human Gene Mutation Database (HGMD), the *CLN5* gene harbors a diverse range of variants: 23 missense (38.98%), 13 nonsense (22.03%), 3 regulatory (5.08%), 3 large and 11 small deletions (18.6%), and 3 small insertions (5.08%) (Figure 4C). A 25-base pair duplication (c.1116_1140) and a splice-site variant have also been documented (29) (Figure 4C). A summary of clinical data from previously reported cases is presented in Table 3.

**Table 3:** Variants and clinical characteristics of selected patients according to literature data.

Patient/Sex	Variants of the *CLN5* gene	Age at disease onset (y mo)	Age at symptom onset (y mo)						Age at death (y mo)	EEG	References
			Learning/ cognition	Motor	Language	Behavior	Vision	Seizures			
Our case/M	Hom p.Arg123Lysfs*4	4	4	Ataxia since 6 y. No independent walking since 8 y 2 mo.	4	4 y 2 mo	normal	6	Alive	7 y 6 mo disorganized cortical activity with prominent epileptiform features	Our case
1/M	Hom p.Gly128Trpfs*10	5	5 y 6 mo	6 y 6 mo	5 y 6 mo	5	5	5	Alive	Not known	(25)
2/F	Hom p.Arg150*	4	5	5	5	5	5	4	Alive	Not known	
3/F	Hom p.Trp175*	4	Yes	4 y 6 mo	4	9	10	9	Alive	Not known	
4/M	p.Trp175* p.Val214Glu	7	7	10	7	9 y 6 mo	9 y 6 mo	8 y 6 mo	Alive	Not known	
5/F	p.Tyr209Asp p.Trp330*	6	6	6	6	12	10	8	20	Not known	
6/M	Hom p.Trp330*	5	6	5	5	No	5	6	Alive	Not known	
7/F	Hom p.Arg63His	3	8	3 y 6 mo	3	9	8	9	Alive	Not known	
8/M	p.Arg63His p.Trp158Arg	2	5	7 y 6 mo	7 y 6 mo	2	6	7 y 6 mo	16 y 6 mo	Not known	
9/M	p.Arg63His p.Trp158Arg	3	4	7	2	3	7	7	14	Not known	
10/M	Hom p.Tyr209Asp	7	7	8	8	7	10 y 6 mo	10 y 6 mo	Alive	Not known	
11/M	Hom p.Tyr209Asp	5	10	13	10	5	10	13	Alive	Not known	
12/M	Hom p.Tyr343*	4	4	6 y 6 mo	8	Not known	6	8	23 y 2 mo	Not known	
13/F	Hom p.Tyr343*	7 y 6 mo	7 y 6 mo	7 y 6 mo	11	Not known	8	10	22 y 1 mo	Not known	
14/F	Hom p.Tyr343*	5	5	8	8 y 6 mo	Not known	8 y 6 mo	8 y 6 mo	20 y 2 mo	Not known	
15/M	Hom p.Tyr343*	5	5	5	8	8	5 y 6 mo	8	Alive	Not known	
M	Hom p.Tyr343*	3	3	Ataxia since 8 y. No independent walking since 9 y.	Not known	Not known	6	8	Alive	Giant f VEP since 10 y	(31)
M	Hom p.Tyr343*	3	3	Ataxia since 8 y. No independent walking since 9 y.	Not known	Not known	6	8	Alive	Giant f VEP since 6 y	
M	Hom p.Tyr343*	4	6	Ataxia since 9 y. No independent walking since 10 y.	Not known	Not known	6	10	19	Giant f VEP since 8 y	
F	Hom p.Trp26*	3	7	Ataxia since 11 y. No independent walking since 11 y.			6		Alive	Giant f VEP since 8 y	
F	Hom p.Trp26*	4	7	Not known	Not known	Not known	6	Not known	Not known	Not known	
M	Hom p.Asp230Asn	2	3	Ataxia since 7 y. No independent walking since 8 y.	Not known	Not known	5	7	13	The absence of giant VEP at 7 y	
F	p.Glu204* p.Tyr343*	4	7	Ataxia since 8 y. No independent walking since 9 y.	Not known	Not known	7	9	Alive	Giant f VEP since 9 y	
F	p.Glu204* p.Trp26*	3	7	Ataxia since 9 y. No independent walking since 10 y.			8	8	Alive	Giant f VEP since 9 y	
F	Hom p.Arg96Pro	5 y 6 mo	Not known	Ataxia since 6 y. No independent walking since 7 y.	6	Not known	6	5 y 6 mo	Alive	EEG at 5 y 6 mo: prominent delta rhythm, diffuse generalized slow spike, and slow wave.	(27)

In the present case, the patient’s parents are non-consanguineous but belong to the same small ethnic group in Dagestan, suggesting a possible founder effect. The identified *CLN5* variant was found in the CSP FMBA population database at a frequency of 0.0000165615 (observed in two healthy adults aged 30–49 years) (21). Some of the most commonly reported *CLN5* variants, such as p.Tyr343* and p.Trp330*, are not present in the Russian population. The p.Arg150* variant, while absent in the Finnish population, has been identified in individuals of other European descent; however, it has not been reported among residents of the Russian Federation (19, 21).

Currently, there is no standardized treatment protocol for NCL type 5. Anticonvulsant therapy demonstrates considerable variability, with pharmacological responses differing significantly between patients. Some reports suggest the efficacy of lamotrigine, particularly in combination with age-appropriate doses of clonazepam (24). Although complete seizure control was not achieved in this case, a partial reduction in frequency and severity was observed. It is worth noting that sodium channel blockers are generally ineffective for epilepsy associated with NCL disorders.

## 4 Discussion

Thus, this article presents a rare case involving a unique combination of two uncommon conditions: NCL type 5 and cervical lymphangioma. Developmental regression was first observed at the age of 4, with the onset of epileptic seizures occurring at age 6. A definitive diagnosis was established at 7 years and 6 months. EEG monitoring during wakefulness revealed a characteristic pattern, including a generalized atonic seizure with diffuse ictal activity. The most recent MRI findings demonstrated cerebellar atrophy, cerebral subatrophy, and lateral ventricular dilation measuring 14 mm. The prognosis in this case remains poor due to progressive dementia, worsening motor decline, and persistent epilepsy. The presence of lymphangioma, along with tracheostomy dependence and long-term use of sirolimus, also negatively impacts the patient’s condition. Surgical revision of the neck mass and tracheostomy removal are not currently feasible due to the advanced stage of the underlying disease.

The authors highlight the importance of comprehensive diagnostic evaluation in patients presenting with neuropsychological regression, even in the presence of severe comorbid conditions. In this case, earlier initiation of antiepileptic therapy might have mitigated seizure frequency in the initial stages of the disease. However, despite multiple therapeutic attempts, complete seizure control has not been achieved, and the patient is currently classified as palliative. We hope that this case contributes to the development of more effective strategies for the management of epilepsy in patients with neuronal ceroid lipofuscinosis type 5. The authors also believe that the presented literature review holds scientific value by consolidating current knowledge and facilitating further research into the clinical features and treatment approaches for this rare disorder.

## Data Availability

The original contributions presented in the study are included in the article, further inquiries can be directed to the corresponding author.
